# Characterizing Developmental Trajectories and the Role of Neuropsychiatric Genetic Risk Variants in Early-Onset Depression

**DOI:** 10.1001/jamapsychiatry.2018.3338

**Published:** 2018-10-14

**Authors:** Frances Rice, Lucy Riglin, Ajay K. Thapar, Jon Heron, Richard Anney, Michael C. O’Donovan, Anita Thapar

**Affiliations:** 1 Medical Research Council for Neuropsychiatric Genetics and Genomics, Division of Psychological Medicine and Clinical Neurosciences, Cardiff University, Cardiff, United Kingdom; 2 School of Social and Community Medicine, University of Bristol, Bristol, United Kingdom

## Abstract

**Importance:**

Depression often first manifests in adolescence. Thereafter, individual trajectories vary substantially, but it is not known what shapes depression trajectories in youth. Adult studies suggest that genetic risk for schizophrenia, a psychiatric disorder with a neurodevelopmental component, may contribute to an earlier onset of depression.

**Objective:**

To test the hypothesis that there are distinct trajectories of depressive symptoms and that genetic liability for neurodevelopmental psychiatric disorders (eg, schizophrenia, attention deficit/hyperactivity disorder [ADHD]), as well as for major depressive disorder (MDD), contribute to early-onset depression.

**Design, Setting, and Participants:**

The Avon Longitudinal Study of Parents and Children is an ongoing, prospective, longitudinal, population-based cohort that has been collecting data since September 6, 1990, including data on 7543 adolescents with depressive symptoms at multiple time points. The present study was conducted between November 10, 2017, and August 14, 2018.

**Main Outcomes and Measures:**

Trajectories based on self-reported depressive symptoms dichotomized by the clinical cutpoint; MDD, schizophrenia, and ADHD polygenic risk score (PRS) were predictors.

**Results:**

In 7543 adolescents with depression data on more than 1 assessment point between a mean (SD) age of 10.64 (0.25) years and 18.65 (0.49) years (3568 [47.3%] male; 3975 [52.7%] female), 3 trajectory classes were identified: persistently low (73.7%), later-adolescence onset (17.3%), and early-adolescence onset (9.0%). The later-adolescence–onset class was associated with MDD genetic risk only (MDD PRS: odds ratio [OR], 1.27; 95% CI, 1.09-1.48; *P* = .003). The early-adolescence–onset class was also associated with MDD genetic risk (MDD PRS: OR, 1.24; 95% CI, 1.06-1.46; *P* = .007) but additionally with genetic risk for neurodevelopmental disorders (schizophrenia PRS: OR, 1.22; 95% CI, 1.04-1.43; *P* = .01; ADHD PRS: OR, 1.32; 95% CI, 1.13-1.54; *P* < .001) and childhood ADHD (χ^2^_1_ = 6.837; *P* = .009) and neurodevelopmental traits (pragmatic language difficulties: OR, 1.31; *P* = .004; social communication difficulties: OR, 0.68; *P* < .001).

**Conclusions and Relevance:**

The findings of this study appear to demonstrate evidence of distinct depressive trajectories, primarily distinguished by age at onset. The more typical depression trajectory with onset of clinically significant symptoms at age 16 years was associated with MDD genetic risk. The less-common depression trajectory, with a very early onset, was particularly associated with ADHD and schizophrenia genetic risk and, phenotypically, with childhood ADHD and neurodevelopmental traits. Findings are consistent with emerging evidence for a neurodevelopmental component in some cases of depression and suggest that the presence of this component may be more likely when the onset of depression is very early.

## Key Points

**Question:**

Do neuropsychiatric disorder genetic risk variants influence developmental trajectories of depression in youth?

**Findings:**

In this population-based study including 7543 adolescents, distinct depression trajectory classes were identified. A later-adolescence–onset class (17.3% of the sample) showed a typical depression trajectory and was associated with major depressive disorder risk alleles, and an early-adolescence–onset class (9.0%) showed clinically significant symptoms at age 12 years and was associated with schizophrenia and attention-deficit hyperactivity disorder genetic risk, childhood attention-deficit hyperactivity disorder, and neurodevelopmental traits.

**Meaning:**

Depression in youth is heterogeneous; findings are consistent with emerging evidence for a neurodevelopmental component to some cases of depression and that this component is more likely when onset is very early.

## Teaser

This population-based study examines the association between genetic risk variants for neuropsychiatric disorders and the age at onset and trajectories of depression in children.

## Introduction

Major depressive disorder (MDD) is the most common mental disorder and a leading cause of disability^1^; even subthreshold depressive symptoms are associated with functional impairment and future mental health problems.^2,3^ Depression often first manifests in adolescence^4,5,6^ and, thereafter, individual trajectories of depressive symptoms vary substantially.^7^ A family history of depression and an early age at onset are each associated with a more chronic symptom course in adults with MDD,^8,9,10^ but it is not known what shapes early depression trajectories in youth.

Depression has a complex multifactorial set of causes, including a moderate heritable component.^4,11,12^ Longitudinal and family studies show continuity between both adolescence-onset depressive disorder and symptoms with depression in adult life, but there are also developmental differences between depression in children, adolescents, and adults.^4^ For instance, clinical follow-up studies of very early-onset depression (average age at onset, 10.7 years) report high rates of heterotypic continuity, where depression is often followed by a different type of clinical disorder.^13,14,15^ Twin studies also show differences in the genetic set of causes of very early-onset depressive symptoms compared with those arising in mid- to late adolescence.^16,17,18^

At the molecular level, a recent genome-wide association study of adults with MDD found evidence of differences in the genetic architecture of depression where a relatively early age at onset (before the median age at onset of 27 years) was associated with genetic liability to schizophrenia, an association not seen for later-onset depression, which was instead associated with MDD risk alleles.^19^ Similar findings have been reported for emotional problems (symptoms of depression and anxiety) in that emotional problems in childhood were associated with schizophrenia risk alleles, but in adult life they were additionally associated with MDD genetic risk.^20^ The association of schizophrenia risk alleles with childhood emotional problems was particularly pronounced in those with emotional problems in both childhood and adulthood, suggesting that persistent emotional symptoms beginning early may drive the association with schizophrenia risk alleles. As schizophrenia genetic risk is thought to involve an early neurodevelopmental component,^21,22^ the role of genetic risk for other neurodevelopmental disorders in early-onset depression may be important to consider. In particular, genetic risk for ADHD, a common childhood-onset neurodevelopmental disorder, may be important in early-onset depression because cross-sectional and longitudinal cohort studies show heightened rates of depression in children with ADHD,^23,24,25^ which may be partly due to the strong genetic correlation between ADHD and depression (*r_g_* = 0.424).^26,27^

Herein, we test the contribution of neuropsychiatric disorder genetic risk variants, specifically genetic liability to MDD, schizophrenia, and ADHD, to early depression trajectories. Schizophrenia and ADHD were selected in addition to MDD as they show moderate to high genetic correlations with major depression,^27^ there is evidence linking schizophrenia polygenic risk score (PRS) to early-onset depression,^19,20^ and epidemiologic and clinical evidence^15,23,24,25^ that ADHD may be an important antecedent of depression.

Estimates of genetic liability to the disorders in the form of PRSs were derived from risk alleles defined in the largest available genome-wide association study of those disorders.^26,28,29^ We did not have a specific hypothesis for bipolar disorder genetic risk because existing studies reporting conflicting results about the phenotypic association between early-onset depression and bipolar disorder,^13,15,30^ with little evidence that this association is stronger for early-onset depression. Bipolar disorder also differs from ADHD and schizophrenia in that evidence suggests that bipolar disorder is less neurodevelopmental in origin.^21,22^ However, for completeness, we included bipolar PRSs in our analyses (eTable 2A in the Supplement). We hypothesized that ADHD and schizophrenia genetic risk would show an association with early-onset depression and that depression genetic risk would be associated with depression with an onset later in adolescence.

## Methods

The Avon Longitudinal Study of Parents and Children (ALSPAC) is an ongoing, population-based, prospective, longitudinal UK birth cohort.^31,32^ Data collection began September 6, 1990. The enrolled core sample consisted of 14 541 pregnant women living in Avon, England, with expected delivery dates between April 1, 1991, and December 31, 1992. Of these births, 13 988 children were alive at 1 year. An additional 713 children who would have been eligible but whose mothers did not choose to participate during pregnancy were enrolled after age 7 years, giving a total sample of 14 701 children alive at 1 year. The present study was conducted between November 10, 2017, and August 14, 2018.

Ethical approval for the study was obtained from the ALSPAC Ethics and Law Committee and the local research ethics committees. All participants provided written informed consent; there was no financial compensation.

The study website contains details of all of the data that are available through a fully searchable data dictionary.^33^ For families with multiple births, we included the oldest sibling. Individuals were included in analyses when data on the primary outcome of depressive symptoms were available for at least 2 time points (n = 7543). The sample mean age (SD) was 10.64 (0.25) years at baseline and 18.65 (0.49) years at the final time point. The numbers of individuals with data available at different times are shown in the eFigure in the Supplement.

Depressive symptoms were reported by the young person at 6 time points (ages 10.5, 12.5, 13.5, 16.5, 17.5, and 18.5 years) on the short Mood and Feelings Questionnaire. This is a well-validated symptom checklist^34,35,36^ that includes 13 items about mood symptoms during the past 2 weeks (rated 0, not true; 1, sometimes true; or 2, true; score range, 0-26). Scores above 11 represent clinically significant symptoms,^34,36^ and we analyzed individuals scoring above and below this level to examine trajectories of clinically significant symptoms.

Polygenic risk scores for MDD, schizophrenia, and ADHD were generated in study individuals as the standardized mean number of disorder risk alleles in approximate linkage equilibrium (*R*^2^<0.20), weighted by genome-wide association study allele effect size derived from data of imputed autosomal single-nucleotide polymorphisms. All analyses were performed using Stata, version 13.0 (StataCorp) to implement the PLINK toolset (http://zzz.bwh.harvard.edu/plink/; code is available at https://github.com/ricanney/stata). In brief, best-guess genotype underwent additional marker and individual quality control. Individuals were excluded on the basis of excessive heterozygosity (>4 SDs from sample mean), relatedness (>3 SDs from sample mean), and genotype missingness (>2%). Markers were excluded if they were rare (minor allele count <5), had high levels of missingness (>2%), or deviated from Hardy-Weinberg equilibrium (*P* ≤ 10^−10^) or from reference minor allele frequency (>10%) (eMethods in the Supplement).

Scores were derived from MDD, ADHD, and schizophrenia weights for 152 536, 103 041, and 27 336 single-nucleotide polymorphisms, respectively. Risk alleles were defined as those associated with case status in the most recent Psychiatric Genomics Consortium analyses of MDD, ADHD, and schizophrenia at a threshold of *P* < .50 for depression and ADHD and *P* < .05 for schizophrenia, as these thresholds maximally capture phenotypic variance.^26,27,28,29,37^ Genome-wide association study discovery sample sizes were 130 664 cases and 330 470 controls for MDD, 20 183 cases and 35 191 controls for ADHD, and 35 476 cases and 46 839 controls for schizophrenia. All PRSs were standardized prior to analysis so odds ratios (ORs) represent 1 SD change (eTable 2A in the Supplement for bipolar PRSs). Phenotypic measures of neurodevelopmental problems (*DSM-IV*^38^ diagnoses of childhood ADHD, social communication problems, and pragmatic language difficulties at age 7 years), psychotic experiences (ages 12 and 17 years), family history of severe depression and schizophrenia, and maternal educational level were used (eMethods in the Supplement).

### Statistical Analysis

We characterized depression trajectories of symptoms dichotomized by clinical cutpoint (n = 7543) using latent class growth analysis in Mplus, version 8.^39^ This analysis is a probability-based technique used to identify an optimum number of distinct patterns (classes) of growth (change) in the longitudinal depression scores of individuals.^40^ Models were run with increasing numbers of classes, starting with a 1-class solution specifying both linear and quadratic change with 500 random starting values and 50 optimizations. Residual variances were allowed to vary across measurement points. A maximum likelihood parameter estimator for which SEs are robust to nonnormality was used.

To examine associations with categorical variables (eg, sex), the DCAT auxiliary option in MPlus was used. A bias-free, 3-step approach in MPlus (R3STEP) estimated the associations between continuous hypothesized predictor variables (PRSs) and trajectory class.^41,42,43^ Model selection was informed by model fit indices and interpretability as recommended.^44^ Full information maximum likelihood estimation was used in MPlus and included all individuals with more than 1 depression assessment in analyses (eTable 1 in the Supplement). For tests of PRS association with trajectory class, we reran analyses using inverse probability weighting^45^ to address any potential bias caused by participant dropout. The pattern of results was similar (eTable 3 in the Supplement).

## Results

### Depression Symptom Trajectories

A 3-class trajectory model provided the best fit to the data and results that were most readily interpretable (eTable 1 and eAppendix 1 in the Supplement). The Figure shows the 3 distinct trajectory classes: a persistently low class (73.7%), a later-adolescence–onset class (17.3%), and an early-adolescence–onset class (9.0%). In the early-adolescence–onset class, the probability of clinically significant depression was first elevated (as indicated by a probability of clinically significant depression symptoms of 0.44) at age 12.5 years, which rose to 0.52 at 13.5 years. In the later-adolescence–onset class, the probability of clinically significant depression (probability, 0.47) was first elevated at age 16.5 years and rose at 17.5 years (probability, 0.57). Both elevated trajectories were associated with a diagnosis of MDD (assessed by the Clinical Interview Schedule–Revised^46^ at age 17.5 years) providing validation of the trajectory classes (later-adolescence onset, 34.4%; early-adolescence onset, 22.8%; low level, 1.5%; overall difference, χ^2^_2_ = 193.70; *P* = .001). The estimated proportion of females was 45.8% in the low-level class and was higher, but did not differ significantly, between the early-adolescence– (74.3%) and later-adolescence– (73.2%) onset classes (Table 1).

**Figure.**
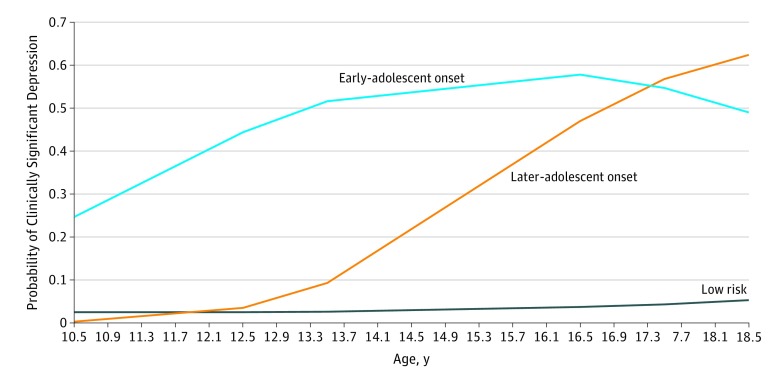
Developmental Trajectories of Depressive Symptoms Depression trajectories identified by latent class growth analyses.

**Table 1.** Phenotypic Associations With Trajectory Class^a^

Variable	Onset, OR (95% CI)				Difference Between Early- and Later-Adolescence–Onset Classes^b^	
	Early Adolescence	*P* Value	Later Adolescence	*P* Value	χ^2^_1_ or OR (95% CI)	*P* Value
Sex, %	74.3	<.001	73.2	<.001	χ^2^_1_ = 0.015	.90
Maternal education, completed A-levels, %^c^	39.1	.01	34.9	.001	χ^2^_1_ = 0.707	.44
Childhood ADHD, %	6.3	.008	0.9	.37	χ^2^_1_ = 6.837	.009
Pragmatic language difficulties^d^	0.63 (0.55-0.71)	<.001	0.82 (0.72-0.94)	.006	OR, 1.31	.004
					χ^2^_1_ = 11.709	.001 (for Cutpoint)
Social communication difficulties^e^	1.50 (1.34-1.68)	<.001	1.01 (0.87-1.18)	.86	OR, 0.68	<.001
					χ^2^_1_ = 18.819	.001 (for Cutpoint)
Psychotic experiences						
12 y	1.47 (1.35-1.61)	<.001	0.89 (0.64-1.22)	.46	OR, 0.60	.003
17 y	1.57 (1.36-1.80)	<.001	1.54 (1.33-1.79)	<.001	OR, 0.99	.74

Abbreviations: ADHD, attention deficit/hyperactivity disorder; OR, odds ratio.

^a^ Continuous scores are standardized so that ORs are for 1-SD increase. Low-risk group was the reference group except for tests of comparison between early-adolescence– and later-adolescence–onset groups where the early-adolescence–onset group was the reference group.

^b^ χ^2^ Tests of difference for social communication and pragmatic language difficulties used the established clinical cut-points for identifying problems (eAppendix in the Supplement). The OR values represent the difference between the ORs in the preceding columns for later-adolescence onset vs early-adolescence onset.

^c^ A-level education is equivalent to high school diploma in the United States

^d^ Lower scores represent more difficulties.

^e^ Higher scores represent more problems.

### Neuropsychiatric PRS and Trajectory Class

As reported in Table 2, the later-adolescence–onset class was associated only with higher MDD PRS (OR, 1.27; 95% CI, 1.09-1.48; *P* = .003). The early-adolescence–onset class was associated with higher PRSs for ADHD (OR, 1.32; 95% CI, 1.13-1.54; *P* < .001), schizophrenia (OR, 1.22; 95% CI, 1.04-1.43; *P* = .01), and MDD (OR, 1.24; 95% CI, 1.06-1.46; *P* = .007). Post hoc, we examined the association with all 3 psychiatric PRSs and trajectory class to examine which PRS contributed most strongly (Table 2). As expected, the PRSs were correlated (eTable 2B and C in the Supplement).

**Table 2.** Associations of Polygenic Risk Scores With Trajectory Classes

Association	Onset, OR (95% CI)			
	Early Adolescence	*P* Value	Later Adolescence	*P* Value
Univariate				
MDD PRS	1.24 (1.06-1.46)	.007	1.27 (1.09-1.48)	.003
Schizophrenia PRS	1.22 (1.04-1.43)	.01	.95 (0.82-1.11)	.56
ADHD PRS	1.32 (1.13-1.54)	<.001	.94 (0.80-1.11)	.48
Multivariate				
MDD PRS	1.16 (0.98-1.36)	.09	1.31 (1.12-1.53)	.001
Schizophrenia PRS	1.19 (1.01-1.41)	.04	.93 (0.79-1.10)	.39
ADHD PRS	1.27 (1.08-1.50)	.003	.90 (0.76-1.07)	.23

Abbreviations: ADHD, attention deficit/hyperactivity disorder; MDD, major depressive disorder; OR, odds ratio; PRS, polygenic risk score.

Multivariate analysis showed that the strongest association with the early-adolescence–onset class was observed for ADHD PRS, the association with schizophrenia PRS was retained, and the association with MDD PRS became nonsignificant (Table 2). Results for the later-adolescence–onset class remained the same. Comparing the early- and later-onset classes showed significant differences (MDD PRS: OR, 1.13; 95% CI, 0.88-1.46; *P* = .33; schizophrenia PRS: OR, 0.78; 95% CI, 0.60-1.01; *P* = .08; and ADHD PRS: OR, 0.71; 95% CI, 0.55-0.92; *P* = .009). Bipolar PRS was not associated with trajectory classes (eTable 2A in the Supplement). Including ancestry-derived principal components did not alter the results (eAppendix 2 in the Supplement).

We tested whether the trajectory classes differed phenotypically on traits conceptually related to ADHD PRS (childhood ADHD and neurodevelopmental traits) and schizophrenia PRS (psychotic experiences). For childhood neurodevelopmental traits, there is evidence that these traits are associated with both ADHD and ADHD PRS^47,48^; for psychotic experiences, there is inconsistent evidence that these experiences are linked with psychosis and schizophrenia PRSs^49,50^ (Table 1). Individuals in the early-adolescence–onset class had higher rates of childhood ADHD (6.3%) than the later-adolescence–onset (0.9%) and low (1.7%) classes and more social communication and pragmatic language problems (Table 1). Proportions scoring above the standard cut points were early onset, 20.7%; later onset, 4.2%; and low level, 5.8% for social communication and early onset, 13.3%; later onset, 2.1%; and low level, 1.4% for pragmatic language. These differences distinguished the early-adolescence– and later-adolescence–onset classes (Table 2). For psychotic experiences, these differences distinguished the early-adolescence– and later-adolescence–onset classes only at age 12 years.

## Discussion

This study identified variation in the developmental trajectories of depression from childhood to early adult life, and moreover, that this variation is partly attributable to MDD, schizophrenia, and ADHD risk alleles. We found evidence of distinct depressive trajectories primarily distinguished by age at onset. We found that the more common, typical developmental trajectory, with onset after puberty and persistence into early adulthood,^6,51^ was associated with elevated genetic risk for depression indexed by MDD PRS. In contrast, we found that depressive symptoms defined by a very early onset (by age 12 years) were associated with all neuropsychiatric genetic risk scores assessed, with multivariate analysis showing that the association was strongest for ADHD PRS.

 Phenotypically, childhood neurodevelopmental difficulties (ADHD, pragmatic language, and social communication difficulties) differentiated the depression trajectories that were elevated only in the early-adolescence–onset group with rates increased by 5- to 7-fold in the early-adolescence–onset group. Psychotic experiences differentiated the groups only at age 12 years. This discrepancy may be driven by depressive symptom differences between the groups at age 12 years (Figure) given the reported association between psychotic experiences and depression and an inconsistent association with psychotic experiences and schizophrenia PRS.^49,50^ The findings are consistent with a growing body of literature showing that depression has heterogeneous causes partly indexed by age at onset. In particular, studies of adult MDD and symptoms measured continuously in population-based samples illustrate that a relatively earlier onset is more strongly associated with schizophrenia polygenic risk.^19,20,52^ We found an additional contribution from ADHD PRSs.

The implication of those results is that early- and later-adolescence-onset depression differ to some extent with respect to the risk factors involved and that the earlier-onset disorder is more strongly influenced by neurodevelopmental factors than depression with a more typical onset in later adolescence or early adulthood. This finding is consistent with a number of observations from epidemiologic, family, and clinical studies. First, several family and clinical follow-up studies suggest that childhood-onset depression might differ etiologically from adolescent-onset depression.^53,54,55,56^ Second, the epidemiologic factors associated with very early-onset depression differ from those of depression with onset in midadolescence to late adolescence in the sex ratio of affected individuals and long-term psychiatric outcomes.^13,57^ Third, neurodevelopmental difficulties, including speech abnormalities and poor motor skills, are particularly associated with early-onset rather than adolescent- or adult-onset depression.^15,58,59^ Fourth, substantial clinical evidence shows that children with ADHD, a common neurodevelopmental disorder, are at elevated risk of subsequent depressive symptoms, suicide attempt, and emotional problems in adult life.^25,60,61,62,63^

Theory suggests neurodevelopmental difficulties as one route to emotional disturbance through the repeated experience of academic failure and peer rejection,^64^ although ADHD and depression may also be associated owing to common risk factors.^65^ A clinical issue is that the response to antidepressant medication^66,67,68,69^ in youth is not as good as it is in adults and evidence suggests the response to tricyclic antidepressants may differ in prepubertal vs postpubertal depression. One possibility is that more neurodevelopmental depression shows a different type of treatment response.

The present study indicates that genetic risk for ADHD and schizophrenia in the general population is associated with a persistent, early-onset trajectory of depressive symptoms. Such effects could operate through overlapping biological pathways as well as evocative gene-environment correlation where genetic factors influence traits that then affect environmental exposures (eg, social exclusion) associated with depression. Irritability, which is common in children with ADHD and other neurodevelopmental disorders, is indexed by genetic risk for ADHD in youth^70^ and has been shown to increase risk for later depression,^71,72^ may be a potential route through which ADHD genetic risk increases the likelihood of mood problems.

Among those with early-onset depression, we did not identify the equal sex ratio of affected males and females that has often been reported when depression onset is very early.^4,73^ This finding was somewhat surprising, and several factors may have contributed to it. First, some research suggests that depression is particularly likely in females with neurodevelopmental disorders, which may imply that high neurodevelopmental risk is more likely to manifest as mood disorder in females.^24,48,74^ Second, while it is generally accepted that self-reports of adolescent mood (as used in the present study) are reliable, children with neurodevelopmental disorders, predominately boys, may underreport their mood symptoms compared with typically developing children.^75^ This reporting difference raises the possibility that the reliance on self-reported mood necessary in the present study due to repeated longitudinal assessments (see below) may mean that some individuals at high neurodevelopmental risk may have been misclassified. Finally, PRSs alone are unlikely to be able to reliably classify children’s risk of developing different types of depression trajectories. However, collectively, results converge to suggest that neurodevelopmental phenotypes (ADHD, as well as social communication and pragmatic language difficulties) and neurodevelopmental genetic risk indicate a greater probability of an early-onset depression trajectory. Phenotypic childhood neurodevelopmental problems were markedly increased in the early-adolescence–onset group (by 5- to 7-fold) compared with the typical depression trajectory. Studies with follow-up further into adult life will help to clarify the adult mental health outcomes of these groups.

### Strengths and Limitations

Strengths of this study include the repeated-measures longitudinal design where depression was assessed using the same measure and informant. Typically, longitudinal studies include changes in measurement and informant, in particular, as children age, the informant tends to change from the parent to the young person. This variation provides a challenge to studies seeking to examine the development of symptoms over time because changes of measurement and informant can affect results. This invariance of measurement over time is a strength.

One limitation of our investigation is that, like many longitudinal studies, nonrandom attrition occurs in ALSPAC over time (eAppendix 2 in the Supplement). This nonrandom loss of participants is likely to result in conservative estimates of the prevalence of the elevated depression trajectory groups. We used a number of approaches to deal with missing data, including full information maximum likelihood in trajectory modeling and inverse probability weighting for tests of association. The pattern of results was replicated using inverse probability weighting. Nonetheless, the missing data assumption made in our analyses is that there are not systematic differences between participants who do and do not provide trajectory data and membership in the sample after conditioning on the other variables in the model (eg, PRS and variables included in the inverse probability weighting analysis).

Depression was assessed using self-reported questionnaires rather than clinical assessment. Nonetheless, subthreshold symptoms are associated with impairment and subsequent MDD.^2,3,4^ It was not possible to investigate rates of mania or bipolar disorder in the trajectory groups. However, evidence is inconsistent on the link with early-onset depression and bipolar disorder.^13,15,30^ The follow-up period in this study was limited to early adult life. A final limitation is that PRSs are weak predictors and explain only a small to modest proportion of phenotypic variance as they do in the present article. However, they provide a useful biological indicator of genetic liability.^76^

## Conclusions

The findings of this study suggest etiologically distinct trajectories of depressive symptoms in youth dependent on age at onset. The findings also show that neurodevelopmental genetic risk contributes to very early-onset depression.

## Notes

Supplement. **eMethods.** Other Assessments **eFigure.** Number of Individuals With Data Available at Each Measurement Point **eTable 1.** Model Fit Indices for Latent Class Growth Models of Self-reported Depression **eAppendix 1.** Additional Analyses **eTable 2A.** Associations Between Bipolar PRS and Trajectory Class **eTable 2B.** Correlations Between Polygenic Risk Scores **eTable 2C.** Correlations Between Polygenic Risk Scores and Parent-Reported Family History of Psychiatric Disorder **eAppendix 2.** Additional Analyses **eTable 3.** Associations of Polygenic Risk Scores With Trajectory Classes Using Inverse Probability Weighting

## References

[1] Vos T, Flaxman AD, Naghavi M, Years lived with disability (YLDs) for 1160 sequelae of 289 diseases and injuries 1990-2010: a systematic analysis for the Global Burden of Disease Study 2010. Lancet. 2012;380(9859):2163-2196. doi:10.1016/S0140-6736(12)61729-2 23245607 PMC6350784

[2] Angold A, Costello EJ, Farmer EM, Burns BJ, Erkanli A Impaired but undiagnosed. J Am Acad Child Adolesc Psychiatry. 1999;38(2):129-137. doi:10.1097/00004583-199902000-00011 9951211

[3] Johnson J, Weissman MM, Klerman GL Service utilization and social morbidity associated with depressive symptoms in the community. JAMA. 1992;267(11):1478-1483. doi:10.1001/jama.1992.03480110054033 1538538

[4] Thapar A, Collishaw S, Pine DS, Thapar AK Depression in adolescence. Lancet. 2012;379(9820):1056-1067. doi:10.1016/S0140-6736(11)60871-4 22305766 PMC3488279

[5] Rohde P, Lewinsohn PM, Klein DN, Seeley JR, Gau JM Key characteristics of major depressive disorder occurring in childhood, adolescence, emerging adulthood, adulthood. Clin Psychol Sci. 2013;1(1). doi:10.1177/2167702612457599 24273703 PMC3833676

[6] Weissman MM, Wickramaratne P, Nomura Y, Warner V, Pilowsky D, Verdeli H Offspring of depressed parents: 20 years later. Am J Psychiatry. 2006;163(6):1001-1008. doi:10.1176/ajp.2006.163.6.1001 16741200

[7] Patton GC, Coffey C, Romaniuk H, The prognosis of common mental disorders in adolescents: a 14-year prospective cohort study. Lancet. 2014;383(9926):1404-1411. doi:10.1016/S0140-6736(13)62116-9 24439298

[8] Musliner KL, Trabjerg BB, Waltoft BL, Parental history of psychiatric diagnoses and unipolar depression: a Danish National Register-based cohort study. Psychol Med. 2015;45(13):2781-2791. doi:10.1017/S0033291715000744 25920726 PMC4746718

[9] Lieb R, Isensee B, Höfler M, Pfister H, Wittchen HU Parental major depression and the risk of depression and other mental disorders in offspring: a prospective-longitudinal community study. Arch Gen Psychiatry. 2002;59(4):365-374. doi:10.1001/archpsyc.59.4.365 11926937

[10] Rhebergen D, Lamers F, Spijker J, de Graaf R, Beekman AT, Penninx BW Course trajectories of unipolar depressive disorders identified by latent class growth analysis. Psychol Med. 2012;42(7):1383-1396. doi:10.1017/S0033291711002509 22053816

[11] Rice F, Harold G, Thapar A The genetic aetiology of childhood depression: a review. J Child Psychol Psychiatry. 2002;43(1):65-79. doi:10.1111/1469-7610.00004 11848337

[12] Flint J, Kendler KS The genetics of major depression. Neuron. 2014;81(3):484-503. doi:10.1016/j.neuron.2014.01.027 24507187 PMC3919201

[13] Harrington R, Rutter M, Weissman M, Psychiatric disorders in the relatives of depressed probands: I—comparison of prepubertal, adolescent and early adult onset cases. J Affect Disord. 1997;42(1):9-22. doi:10.1016/S0165-0327(96)00091-2 9089054

[14] Rutter M, Kim-Cohen J, Maughan B Continuities and discontinuities in psychopathology between childhood and adult life. J Child Psychol Psychiatry. 2006;47(3-4):276-295. doi:10.1111/j.1469-7610.2006.01614.x 16492260

[15] Jaffee SR, Moffitt TE, Caspi A, Fombonne E, Poulton R, Martin J Differences in early childhood risk factors for juvenile-onset and adult-onset depression. Arch Gen Psychiatry. 2002;59(3):215-222. doi:10.1001/archpsyc.59.3.215 11879158

[16] Eaves LJ, Silberg JL, Meyer JM, Genetics and developmental psychopathology: 2—the main effects of genes and environment on behavioral problems in the Virginia Twin Study of Adolescent Behavioral Development. J Child Psychol Psychiatry. 1997;38(8):965-980. doi:10.1111/j.1469-7610.1997.tb01614.x 9413795

[17] Thapar A, McGuffin P A twin study of depressive symptoms in childhood. Br J Psychiatry. 1994;165(2):259-265. doi:10.1192/bjp.165.2.259 7953041

[18] Rice F, Harold GT, Thapar A Assessing the effects of age, sex and shared environment on the genetic aetiology of depression in childhood and adolescence. J Child Psychol Psychiatry. 2002;43(8):1039-1051. doi:10.1111/1469-7610.00231 12455925

[19] Power RA, Tansey KE, Buttenschøn HN, ; CONVERGE Consortium, CARDIoGRAM Consortium, GERAD1 Consortium Genome-wide association for major depression through age at onset stratification: Major Depressive Disorder Working Group of the Psychiatric Genomics Consortium. Biol Psychiatry. 2017;81(4):325-335. doi:10.1016/j.biopsych.2016.05.010 27519822 PMC5262436

[20] Riglin L, Collishaw S, Richards A, Schizophrenia risk alleles and neurodevelopmental outcomes in childhood: a population-based cohort study. Lancet Psychiatry. 2017;4(1):57-62. doi:10.1016/S2215-0366(16)30406-0 27932233

[21] Craddock N, Owen MJ The Kraepelinian dichotomy—going, going…but still not gone. Br J Psychiatry. 2010;196(2):92-95. doi:10.1192/bjp.bp.109.073429 20118450 PMC2815936

[22] Owen MJ New approaches to psychiatric diagnostic classification. Neuron. 2014;84(3):564-571. doi:10.1016/j.neuron.2014.10.028 25442935

[23] Kessler RC, Adler LA, Berglund P, The effects of temporally secondary co-morbid mental disorders on the associations of *DSM-IV* ADHD with adverse outcomes in the US National Comorbidity Survey Replication Adolescent Supplement (NCS-A). Psychol Med. 2014;44(8):1779-1792. doi:10.1017/S0033291713002419 24103255 PMC4124915

[24] Avenevoli S, Swendsen J, He JP, Burstein M, Merikangas KR Major depression in the National Comorbidity Survey-Adolescent Supplement: prevalence, correlates, and treatment. J Am Acad Child Adolesc Psych. 2015;54(1):37-44. doi:10.1016/j.jaac.2014.10.010 25524788 PMC4408277

[25] Angold A, Costello EJ, Erkanli A Comorbidity. J Child Psychol Psychiatry. 1999;40(1):57-87. doi:10.1111/1469-7610.00424 10102726

[26] Demontis D, Walters RK, Martin J, Discovery of the first genome-wide significant risk loci for ADHD. Bio Rxiv. https://www.biorxiv.org/content/early/2017/06/03/145581. Published 2017. Accessed January 18, 2018.

[27] Lee SH, Ripke S, Neale BM, ; Cross-Disorder Group of the Psychiatric Genomics Consortium; International Inflammatory Bowel Disease Genetics Consortium (IIBDGC) Genetic relationship between five psychiatric disorders estimated from genome-wide SNPs. Nat Genet. 2013;45(9):984-994. doi:10.1038/ng.2711 23933821 PMC3800159

[28] Schizophrenia Working Group of the Psychiatric Genomics Consortium Biological insights from 108 schizophrenia-associated genetic loci. Nature. 2014;511(7510):421-427. doi:10.1038/nature13595 25056061 PMC4112379

[29] Wray NR, Ripke S, Mattheisen M, ; eQTLGen; 23andMe; Major Depressive Disorder Working Group of the Psychiatric Genomics Consortium Genome-wide association analyses identify 44 risk variants and refine the genetic architecture of major depression. Nat Genet. 2018;50(5):668-681. doi:10.1038/s41588-018-0090-3 29700475 PMC5934326

[30] Kovacs M, Obrosky S, George C The course of major depressive disorder from childhood to young adulthood: recovery and recurrence in a longitudinal observational study. J Affect Disord. 2016;203:374-381. doi:10.1016/j.jad.2016.05.042 27347807 PMC4975998

[31] Boyd A, Golding J, Macleod J, Cohort profile: the “children of the 90s”—the index offspring of the Avon Longitudinal Study of Parents and Children. Int J Epidemiol. 2013;42(1):111-127. doi:10.1093/ije/dys064 22507743 PMC3600618

[32] Fraser A, Macdonald-Wallis C, Tilling K, Cohort profile: the Avon Longitudinal Study of Parents and Children: ALSPAC mothers cohort. Int J Epidemiol. 2013;42(1):97-110. doi:10.1093/ije/dys066 22507742 PMC3600619

[33] University of Bristol Avon Longitudinal Study of Parents and Children. http://www.bristol.ac.uk/alspac/researchers/access/. Accessed August 14, 2108.

[34] Thabrew H, Stasiak K, Bavin LM, Frampton C, Merry S Validation of the Mood and Feelings Questionnaire (MFQ) and Short Mood and Feelings Questionnaire (SMFQ) in New Zealand help-seeking adolescents. Int J Methods Psychiatr Res. 2018;27(3):e1610. doi:10.1002/mpr.1610 29465165 PMC6877137

[35] Costello EJ, Angold A Scales to assess child and adolescent depression: checklists, screens, and nets. J Am Acad Child Adolesc Psychiatry. 1988;27(6):726-737. doi:10.1097/00004583-198811000-00011 3058677

[36] Thapar A, McGuffin P Validity of the shortened Mood and Feelings Questionnaire in a community sample of children and adolescents: a preliminary research note. Psychiatry Res. 1998;81(2):259-268. doi:10.1016/S0165-1781(98)00073-0 9858042

[37] Neale BM, Medland SE, Ripke S, ; Psychiatric GWAS Consortium: ADHD Subgroup Meta-analysis of genome-wide association studies of attention-deficit/hyperactivity disorder. J Am Acad Child Adolesc Psychiatry. 2010;49(9):884-897. doi:10.1016/j.jaac.2010.06.008 20732625 PMC2928252

[38] American Psychiatric Association Diagnostic and Statistical Manual of Mental Disorders. 4th ed, text revision Washington, DC: American Psychiatric Association; 2000.

[39] Muthén LK, Muthén BO MPlus User’s Guide. 7th ed Los Angeles, CA; Muthén & Muthén 2012.

[40] Muthén B, Muthén LK Integrating person-centered and variable-centered analyses: growth mixture modeling with latent trajectory classes. Alcohol Clin Exp Res. 2000;24(6):882-891. doi:10.1111/j.1530-0277.2000.tb02070.x 10888079

[41] Asparouhov TBM Auxiliary variables in mixture modeling: three-step approaches using Mplus. Struct Equ Modeling. 2014;21(3):329-341. doi:10.1080/10705511.2014.915181

[42] Heron JE, Croudace TJ, Barker ED, Tilling KA A comparison of approaches for assessing covariate effects in latent class analysis. Longit Life Course Stud. 2015;6(4):420-434. doi:10.14301/llcs.v6i4.322

[43] van de Schoot R, Sijbrandij M, Winter SD, Depaoli S, Vermunt JK The GRoLTS-Checklist: guidelines for reporting on latent trajectory studies. Struct Equ Modeling. 2017;24(3):451-467. doi:10.1080/10705511.2016.1247646

[44] Berlin KS, Parra GR, Williams NA An introduction to latent variable mixture modeling (part 2): longitudinal latent class growth analysis and growth mixture models. J Pediatr Psychol. 2014;39(2):188-203. doi:10.1093/jpepsy/jst085 24277770

[45] Seaman SR, White IR Review of inverse probability weighting for dealing with missing data. Stat Methods Med Res. 2013;22(3):278-295. doi:10.1177/0962280210395740 21220355

[46] Lewis G, Pelosi AJ, Araya R, Dunn G Measuring psychiatric disorder in the community: a standardized assessment for use by lay interviewers. Psychol Med. 1992;22(2):465-486. doi:10.1017/S0033291700030415 1615114

[47] Riglin L, Collishaw S, Thapar AK, Association of genetic risk variants with attention-deficit/hyperactivity disorder trajectories in the general population. JAMA Psychiatry. 2016;73(12):1285-1292. doi:10.1001/jamapsychiatry.2016.2817 27806167 PMC6485350

[48] Martin J, Hamshere ML, Stergiakouli E, O’Donovan MC, Thapar A Genetic risk for attention-deficit/hyperactivity disorder contributes to neurodevelopmental traits in the general population. Biol Psychiatry. 2014;76(8):664-671. doi:10.1016/j.biopsych.2014.02.013 24673882 PMC4183378

[49] McGrath JJ, Saha S, Al-Hamzawi A, The bidirectional associations between psychotic experiences and *DSM-IV* mental disorders. Am J Psychiatry. 2016;173(10):997-1006. doi:10.1176/appi.ajp.2016.15101293 26988628 PMC5175400

[50] Jones HJ, Stergiakouli E, Tansey KE, Phenotypic manifestation of genetic risk for schizophrenia during adolescence in the general population. JAMA Psychiatry. 2016;73(3):221-228. doi:10.1001/jamapsychiatry.2015.3058 26818099 PMC5024747

[51] Kessler RC, Berglund P, Demler O, Jin R, Merikangas KR, Walters EE Lifetime prevalence and age-of-onset distributions of *DSM-IV* disorders in the National Comorbidity Survey Replication. Arch Gen Psychiatry. 2005;62(6):593-602. doi:10.1001/archpsyc.62.6.593 15939837

[52] Verduijn J, Milaneschi Y, Peyrot WJ, Using clinical characteristics to identify which patients with major depressive disorder have a higher genetic load for three psychiatric disorders. Biol Psychiatry. 2017;81(4):316-324. doi:10.1016/j.biopsych.2016.05.024 27576130

[53] Wickramaratne PJ, Weissman MM Onset of psychopathology in offspring by developmental phase and parental depression. J Am Acad Child Adolesc Psychiatry. 1998;37(9):933-942. doi:10.1097/00004583-199809000-00013 9841243

[54] Harrington R Adolescent depression: same or different? Arch Gen Psychiatry. 2001;58(1):21-22. doi:10.1001/archpsyc.58.1.21 11146754

[55] Weissman MM, Wolk S, Wickramaratne P, Children with prepubertal-onset major depressive disorder and anxiety grown up. Arch Gen Psychiatry. 1999;56(9):794-801. doi:10.1001/archpsyc.56.9.794 12884885

[56] Harrington R, Fudge H, Rutter M, Pickles A, Hill J Adult outcomes of childhood and adolescent depression—I: psychiatric status. Arch Gen Psychiatry. 1990;47(5):465-473. doi:10.1001/archpsyc.1990.01810170065010 2184797

[57] Harrington R, Rutter M, Fombone E Developmental pathways in depression: multiple meanings, antecedents, and endpoints. Dev Psychopathol. 1996;8:601-616. doi:10.1017/S095457940000732X

[58] Sigurdsson E, Van Os J, Fombonne E Are impaired childhood motor skills a risk factor for adolescent anxiety? results from the 1958 UK birth cohort and the National Child Development Study. Am J Psychiatry. 2002;159(6):1044-1046. doi:10.1176/appi.ajp.159.6.1044 12042195

[59] van Os J, Jones P, Lewis G, Wadsworth M, Murray R Developmental precursors of affective illness in a general population birth cohort. Arch Gen Psychiatry. 1997;54(7):625-631. doi:10.1001/archpsyc.1997.01830190049005 9236546

[60] Ljung T, Chen Q, Lichtenstein P, Larsson H Common etiological factors of attention-deficit/hyperactivity disorder and suicidal behavior: a population-based study in Sweden. JAMA Psychiatry. 2014;71(8):958-964. doi:10.1001/jamapsychiatry.2014.363 24964928

[61] Hammerton G, Zammit S, Mahedy L, Pathways to suicide-related behavior in offspring of mothers with depression: the role of offspring psychopathology. J Am Acad Child Adolesc Psychiatry. 2015;54(5):385-393. doi:10.1016/j.jaac.2015.02.006 25901775 PMC4411216

[62] Nock MK, Green JG, Hwang I, Prevalence, correlates, and treatment of lifetime suicidal behavior among adolescents: results from the National Comorbidity Survey Replication Adolescent Supplement. JAMA Psychiatry. 2013;70(3):300-310. doi:10.1001/2013.jamapsychiatry.55 23303463 PMC3886236

[63] Klein RG, Mannuzza S, Olazagasti MA, Clinical and functional outcome of childhood attention-deficit/hyperactivity disorder 33 years later. Arch Gen Psychiatry. 2012;69(12):1295-1303. doi:10.1001/archgenpsychiatry.2012.271 23070149 PMC3597443

[64] Capaldi DM Co-occurrence of conduct problems and depressive symptoms in early adolescent boys—II: a 2 year follow-up at grade 8. Dev Psychopathol. 1992;4:125-144. doi:10.1017/S0954579400005605 10208356

[65] Biederman J, Mick E, Faraone SV Depression in attention deficit hyperactivity disorder (ADHD) children: “true” depression or demoralization? J Affect Disord. 1998;47(1-3):113-122. doi:10.1016/S0165-0327(97)00127-4 9476751

[66] Hazell P, Mirzaie M Tricyclic drugs for depression in children and adolescents. Cochrane Database Syst Rev. 2013;(6):CD002317. 23780719 10.1002/14651858.CD002317.pub2 PMC7093893

[67] Hazell P, O’Connell D, Heathcote D, Robertson J, Henry D Efficacy of tricyclic drugs in treating child and adolescent depression: a meta-analysis. BMJ. 1995;310(6984):897-901. doi:10.1136/bmj.310.6984.897 7719178 PMC2549288

[68] Hetrick SE, McKenzie JE, Cox GR, Simmons MB, Merry SN Newer generation antidepressants for depressive disorders in children and adolescents. Cochrane Database Syst Rev. 2012;11:CD004851. 23152227 10.1002/14651858.CD004851.pub3 PMC8786271

[69] Hetrick SE, McKenzie JE, Merry SN The use of SSRIs in children and adolescents. Curr Opin Psychiatry. 2010;23(1):53-57. doi:10.1097/YCO.0b013e328334bc92 19934760

[70] Riglin L, Eyre O, Cooper M, Investigating the genetic underpinnings of early-life irritability. Transl Psychiatry. 2017;7(9):e1241. doi:10.1038/tp.2017.212 28949337 PMC5639253

[71] Stringaris A, Cohen P, Pine DS, Leibenluft E Adult outcomes of youth irritability: a 20-year prospective community-based study. Am J Psychiatry. 2009;166(9):1048-1054. doi:10.1176/appi.ajp.2009.08121849 19570932 PMC2791884

[72] Rice F, Sellers R, Hammerton G, Antecedents of new-onset major depressive disorder in children and adolescents at high familial risk. JAMA Psychiatry. 2017;74(2):153-160. doi:10.1001/jamapsychiatry.2016.3140 27926743

[73] Maughan B, Collishaw S, Stringaris A Depression in childhood and adolescence. J Can Acad Child Adolesc Psychiatry. 2013;22(1):35-40. 23390431 PMC3565713

[74] Martin J, Walters RK, Demontis D, ; 23andMe Research Team; Psychiatric Genomics Consortium: ADHD Subgroup; iPSYCH–Broad ADHD Workgroup A genetic investigation of sex bias in the prevalence of attention-deficit/hyperactivity disorder. Biol Psychiatry. 2018;83(12):1044-1053. doi:10.1016/j.biopsych.2017.11.026 29325848 PMC5992329

[75] Fraser A, Cooper M, Agha SS, The presentation of depression symptoms in attention-deficit/hyperactivity disorder: comparing child and parent reports. Child Adolesc Ment Health. 2018;23(3):243-250. doi:10.1111/camh.12253 30197576 PMC6120536

[76] Kendler KS The schizophrenia polygenic risk score: to what does it predispose in adolescence? JAMA Psychiatry. 2016;73(3):193-194. doi:10.1001/jamapsychiatry.2015.2964 26817666

