# Supplementary material for: Characterizing Developmental Trajectories and the Role of Neuropsychiatric Genetic Risk Variants in Early-Onset Depression
Source: JAMA Psychiatry. 2018 Oct 14;76(3):306–13. doi: 10.1001/jamapsychiatry.2018.3338 (PMC6439821; doi:10.1001/jamapsychiatry.2018.3338)
Supplement: Supplement. — eMethods. Other Assessments eFigure. Number of Individuals With Data Available at Each Measurement Point eTable 1. Model Fit Indices for Latent Class Growth Models of Self-reported Depression eAppendix 1. Additional Analyses eTable 2A. Associations Between Bipolar PRS and Trajectory Class eTable 2B. Correlations Between Polygenic Risk Scores eTable 2C. Correlations Between Polygenic Risk Scores and Parent-Reported Family History of Psychiatric Disorder eAppendix 2. Additional Analyses eTable 3. Associations of Polygenic Risk Scores With Trajectory Classes Using Inverse Probability Weighting [file jamapsychiatry-76-306-s001.pdf]

Supplementary Online Content

Rice F, Riglin L, Thapar AK, et al. Characterizing developmental trajectories and the role of neuropsychiatric genetic risk variants in early-onset depression. *JAMA Psychiatry*. Published online October 14, 2018. doi:10.1001/jamapsychiatry.2018.3338

eMethods. Other Assessments

eFigure 1. Number of Individuals With Data Available at Each Measurement Point

eTable 1. Model Fit Indices for Latent Class Growth Models of Self-reported Depression

eAppendix 1. Determining the Optimal Number of Classes

eTable 2A. Associations Between Bipolar PRS and Trajectory Class

eTable 2B. Correlations Between Polygenic Risk Scores

eTable 2C. Correlations Between Polygenic Risk Scores and Parent-Reported Family History of Psychiatric Disorder

eAppendix 2. Additional Analyses

eTable 3. Associations of Polygenic Risk Scores With Trajectory Classes Using Inverse Probability Weighting

This supplementary material has been provided by the authors to give readers additional information about their work.

© 2018 Rice F et al. *JAMA Psychiatry.*

eMethods. Other Assessments

Further testing

Social communication problems and pragmatic language difficulties were assessed at age 7 as indicators of childhood neurodevelopmental traits using the parent-reported Social and Communication Disorders Checklist [1]and the pragmatic language subscale of the Children's Communication Checklist[2]. Categorically defined problems were defined using the established cut-points of 9 or over on the Social and Communication Checklist[2] and 132 or less on the pragmatic language sub-scale of the Children's Communication Checklist. A DSM-IV [3] diagnosis of childhood ADHD at age 7 was assessed using parental reports to the Developmental and Wellbeing Assessment (DAWBA) – a widely used, well validated instrument for generating DSM-IV diagnoses of childhood and adolescent neuropsychiatric disorders[4]. Psychotic experiences at age 12 and 17 were assessed with the semi-structured Psychosis-Like Symptom Interview[5,6] which assesses the presence of the psychotic experiences of hallucinations, delusions and thought interference. A total score was calculated (range 0, 12) including only those experiences rated as "definitely present" by trained interviewers[7]. Evidence to date suggests that psychotic experiences are weak predictors of psychotic disorder and are associated with most psychiatric disorders including depression [8,9]. Mothers and fathers reported on their own and their biological parents' current and past history of severe depression and schizophrenia by questionnaire at 12 weeks gestation. Familial loading for depression and schizophrenia was calculated as the number of family members with a history of depression or schizophrenia weighted by relatedness (first or second-degree relative)[10]. Maternal education (assessed during pregnancy) was used as an indicator of socioeconomic position. This was coded as achieved A levels (which is roughly equivalent to a US high school diploma) or a university degree (1) versus did not achieve this level (0).

© 2018 Rice F et al. *JAMA Psychiatry.*

Polygenic risk scores: additional details

Best guess genotype data underwent additional marker and individual quality control. Individuals were excluded based on heterozygosity (greater than 4x standard deviation from sample mean), excessive relatedness (measured by mean kinship against all individuals in the analysis; excessive relatedness was defined as 3x increase standard deviation compared to the sample mean) and genotype missingness (>2%). (These analyses are described in the genotypeqc package at https:github.com/ricanney/stata). Markers were excluded if minor count less than 5, SNP missingness (>2%), Hardy-Weinberg equilibrium ($p \leq 10^{-10}$) and deviation from reference MAF (>10%). Ancestry informative principle components were generated from linkage independent ancestry informative markers using the bim2eigenvec package. Training GWAS for PRS were cleaned using the summaryqc package and processed using summaryqc2prePRS. All markers in training GWAS for PRS were mapped to hg19 and nomenclature standardised to the 1000 genomes reference panel based on chromosome location and IUPAC (International Union of Pure and Applied Chemistry e.g http://www.bioinformatics.org/sms2/iupac.html) genotype code.

Calculating bipolar disorder polygenic risk scores

Scores were derived from bipolar disorder weights for 29,684 SNPs. Risk alleles were defined as those associated with case status in the most recent Psychiatric Genomics Consortium analysis of bipolar disorder[11] at a threshold of $P < .05$ as this threshold maximally capture phenotypic variance[11]. The genome-wide association study discovery sample size was: 20,352 cases and 31,358 controls. The PRS was standardized prior to analysis so odds ratios represent a one standard deviation change.

© 2018 Rice F et al. *JAMA Psychiatry.*

eFigure 1. Number of Individuals With Data Available at Each Measurement Point

A

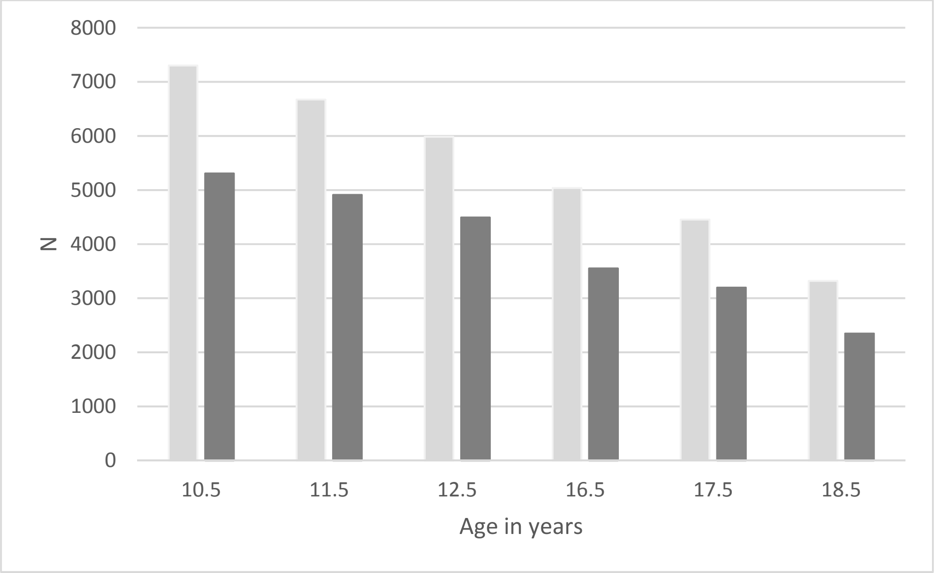

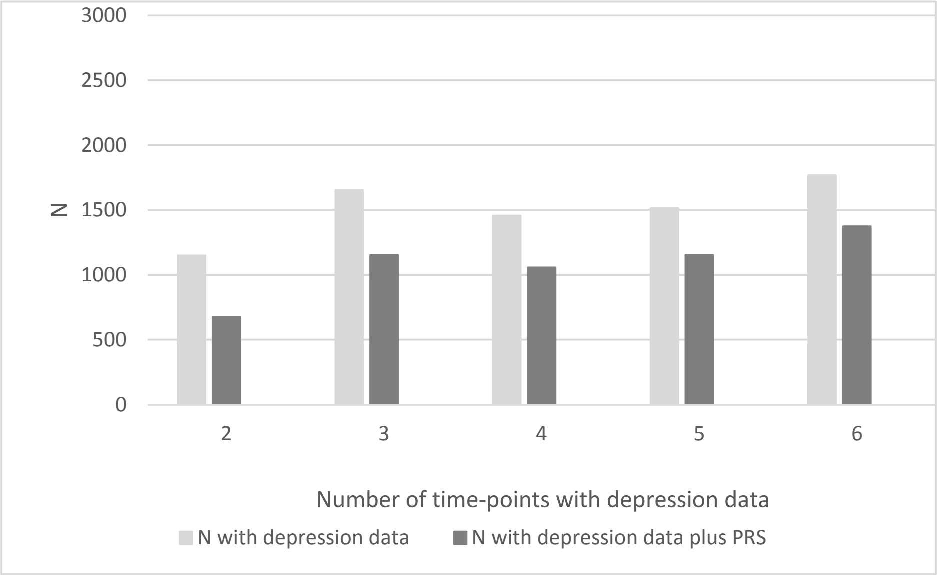

B

Footnote to eFigure 1: of the 7543 individuals with depression data on >1 time point, 5416 had polygenic score data available.

© 2018 Rice F et al. *JAMA Psychiatry.*

eTable 1. Model Fit Indices for Latent Class Growth Models of Self-reported Depression

	Number of classes			
	**2**	**3**	**4**	**5**
Complete measures (6) (n=1769)				
SABIC	6154.66	6120.21	6121.54	6126.52
LL	-3062.28	-3036.45	-3028.51	-3022.40
# parameters	7	11	15	19
Entropy	.709	.788	.802	.774
Smallest class	22%	4%	4%	2%
VLMR-LRT p value	<.001	<.001	.003	<.001
BLRT p value	<.001	<.001	.013	.077
3+ measures (n=6393)				
SABIC	17092.00	16996.61	16973.41	16965.18
LL	-8526.45	-8467.59	-8444.81	-8429.53
# parameters	7	11	15	19
Entropy	.604	.624	.475	0.614
Smallest class	22%	9%	8%	2%
VLMR-LRT p value	<.001	<.001	.007	<.001
BLRT p value	<.001	<.001	<.001	<.001
2+ measures (n=7543)				
SABIC	18612.83	18509.39	18484.79	18476.28
LL	-9286.29	-9223.07	-9199.27	-9183.51
# parameters	7	11	15	19
Entropy	.576	.590	.448	.587
Smallest class	22%	9%	8%	2%
VLMR-LRT p value	<.001	<.001	<.001	.005
BLRT p value	<.001	<.001	<.001	<.001

LL=Loglikelihood; SABIC= sample size adjusted Bayesian Information Criteria; VLMR-LRT=Vuong-Lo-Mendell-Rubin Likelihood Ratio Test; BLRT= Bootstrapped Likelihood Ratio Test. LRT / BLRT tests compare p-class model with p-1 class.

© 2018 Rice F et al. *JAMA Psychiatry.*

eAppendix 1. Determining the Optimal Number of Classes

In line with most recommendations we selected the optimal model on the basis of a number of factors:

1) The meaning, interpretability and face validity of classes based on existing knowledge and theory.
2) Fit indices: the SABIC and the VLMR-LRT have been recommended as the most robust fit indices[12].
3) The size of the smallest class: a minimum of around 5% of the sample.
4) Absence of warnings regarding model fit, and
5) The extent to which the classes replicated when allowing for differing amounts of missing data across the six repeated measurements of depressive symptoms.

The three class model was selected on the basis of these criteria. The three class model included a persistently low class, an early-onset persistent class and a late-adolescent onset class. For the full cases model, there is good evidence for the three class solution based on SABIC. In the models that allow for some missing observations, the SABIC also starts to level out at three classes. We judged the face validity of the three class model to be superior to that of the four class model which included similar classes to those in the three class model with the addition of a persistent moderately low class. The proportions in the smallest class was similar for the models including individuals with two or more and three or more measures of depressive symptoms (9%) but, as might be expected from selective attrition, was lower when restricting the sample to those with complete data. Entropy, a measure of classification uncertainty, was modest-good for the three class solution including those with 2+ and 3+ measures but increased for the full cases models suggesting this is driven by missing data uncertainty.

© 2018 Rice F et al. *JAMA Psychiatry.*

eTable 2A. Associations Between Bipolar PRS and Trajectory Class

	Early-adolescent onset (9.0%) OR	95% CI	p	Late-adolescent onset (17.3%) OR	95% CI	p
Bipolar PRS	1.15	.99, 1.34	.067	1.00	.86, 1.17	.955

eTable 2B. Correlations Between Polygenic Risk Scores

	MDD PRS	Schizophrenia PRS	ADHD PRS
**MDD PRS**	1		
**Schizophrenia PRS**	.146	1	
**ADHD PRS**	.202	.035	1
**Bipolar PRS**	.126	.303	.046

Footnote to eTable 2b: PRS = polygenic risk score. All PRS are standardized. All correlations are significant at p<.01.

eTable 2C. Correlations Between Polygenic Risk Scores and Parent-Reported Family History of Psychiatric Disorder

	Family history of depression (weighted score)	Family history of schizophrenia (weighted score)
**MDD PRS**	.045 ***	-.004
**Schizophrenia PRS**	.025*	.015
**ADHD PRS**	.001	-.005
**Bipolar PRS**	.012	-.012

Footnote to eTable 2c: PRS = polygenic risk score. All PRS are standardized. Number with a positive family history of severe depression in a parent or grandparent = 5067. Number with a positive family history of schizophrenia in a parent or grandparent = 156. *=p<.05; ***= p<.001.

© 2018 Rice F et al. *JAMA Psychiatry.*

eAppendix 2. Additional Analyses

PRS controlling for ancestry principal components

We ran sensitivity analyses including the top 10 principal components. Including principal components in R3STEP tests of association between MDD, schizophrenia and ADHD PRS and trajectory class did not change the pattern of results observed for major depression ("late-onset-adolescent" OR including 10 PCs = 1.27, p=.003; "early-adolescent-onset" OR including 10 PCs = 1.25, p=.008), schizophrenia ("late-onset-adolescent" OR including 10 PCs = .97, p=.667; "early-adolescent-onset" OR including 10 PCs = 1.22, p=.017), or ADHD ("late-onset-adolescent" OR including 10 PCs = .93, p=.400; "early-adolescent-onset" OR including 10 PCs = 1.33, p<0.001). Ancestry derived principal components were therefore not included as covariates or predictor variables in the main analyses reported.

Attrition

To examine the pattern of attrition relevant to the present analyses, we used key variables (self-reported sMFQ depression scores at ages 10.5, 11.5, 12.5, 16.5 and 17.5 years, gender, MDD PRS, schizophrenia PRS and ADHD PRS) to predict missing data status (yes;no) at age 18.5 years in a binary logistic regression. Predictor variables were entered individually. The following variables were associated with a greater likelihood of missing data at age 18.5 years: male gender (OR = .433, p=.001, 95% CI = .399, .469), higher depression symptom score at age 10.5 years (OR=1.023, p=.001, 95% CI = 1.009, 1.037) and at age 17.5 years (OR=1.023, p=.001, 95% CI = 1.012, 1.035); a higher MDD PRS (OR=1.170, p=.001, 95% CI = 1.114, 1.228), a higher schizophrenia PRS (OR = 1.092, p=.001, 95% CI = 1.040, 1.146); a higher ADHD PRS (OR = 1.176, p=.001, 95% CI = 1.120, 1.235). Results were similar when using a linear regression to predict the number of completed data assessments (possible range 0 – 6): A *greater* number of completed assessments was associated with female gender (b=.523, p=.001, 95% CI = .451, .595); fewer depressive symptoms at age 10.5 (b= -.022, p=.001, 95% CI = -.032, -.012), age 16.5 (b= -.012, p=. 002, 95% CI = -.019, -.004) and age 17.5 (b = -.020, p=.001, 95% CI = -.028, -

© 2018 Rice F et al. *JAMA Psychiatry.*

.012); lower MDD PRS (b= -.136, p=.001, 95% CI = -.184, -.008); lower schizophrenia PRS (b= -.158, p=.001, 95% CI = -.206, -.111) and lower ADHD PRS (b= -.220, p=.001, 95% CI = -.267, -.172).

Inverse probability weighting

We used inverse probability weighting (IPW) to investigate the possibility of biased associations due to non-random missingness of genetic data. This approach has been recommended over alternative methods for dealing with missing data (such as multiple imputation) in situations where blocks of data are missing (as is often the case in ALSPAC where missingness of a variable is often due to non-participation in a clinic assessment visit). We also elected to use this approach because it was not valid to impute values for unobserved genetic data. IPW involves weighting complete cases by the inverse probability of their being a complete case and involves specifying a missingness model in order to account for any bias in patterns of association due to missing data[13]. In line with the preliminary analyses above which examined variables predicting missingness and previous publications based on this cohort [14,15], we used data from early time points in the ALSPAC data set on maternal age, parity, partner status and affection (almost always/often vs. sometimes/barely/never), socioeconomic status (A level completion vs. lower education level; financial difficulties), parental psychopathology (depression), child sex and whether or not the parent was in the core ALSPAC sample to predict missingness of PRS data for those included in latent class growth analysis (N=7543, of whom 2127 were missing PRS data). Minimal missing data on indicators used to derive weights were singly imputed as the modal value (all indicators had >6500 values). The Hosmer-Lemeshow test was used assess the fit of the missingness model, with results showing no indication of poor fit (Hosmer-Lemeshow $\chi^2(8)=7.464$, p=0.487). Weights ranged from 1.21 to 1.74. The analyses were re-run using IPW to address any potential bias caused by participant dropout. The pattern of results was very similar (eTable 3).

© 2018 Rice F et al. *JAMA Psychiatry.*

eTable 3. Associations of Polygenic Risk Scores With Trajectory Classes Using Inverse Probability Weighting

	Classes	Early-adolescent-onset (9.0%) OR	95% CI	p	Late-adolescent-onset (17.3%) OR	95% CI	p
Univariate associations	MDD PRS	1.25	1.06, 1.46	.006	1.28	1.10, 1.49	.002
	Schizophrenia PRS	1.22	1.04, 1.43	.016	.95	.81, 1.11	.543
	ADHD PRS	1.32	1.13, 1.54	.001	.94	.80, 1.11	.469
Multivariate associations	MDD PRS	1.16	.98, 1.37	.075	1.32	1.13, 1.55	.001
	Schizophrenia PRS	1.18	1.00, 1.40	.049	.93	.79, 1.09	.376
	ADHD PRS	1.27	1.08, 1.49	.003	.90	.76, 1.06	.212

© 2018 Rice F et al. *JAMA Psychiatry.*

eReferences

1. Skuse DH, Mandy WP, Scourfield J. Measuring autistic traits: heritability, reliability and validity of the Social and Communication Disorders Checklist. *The British journal of psychiatry : the journal of mental science.* 2005;187:568-572.
2. Bishop DV. Development of the Children's Communication Checklist (CCC): a method for assessing qualitative aspects of communicative impairment in children. *Journal of child psychology and psychiatry, and allied disciplines.* 1998;39(6):879-891.
3. APA. *Diagnostic and Statistical Manual of Mental Disorders: DSM-IV-TR.* Washington, DC.: American Psychiatric Press; 2000.
4. Goodman R, Ford T, Richards H, Gatward R, Meltzer H. The Development and Well-Being Assessment: description and initial validation of an integrated assessment of child and adolescent psychopathology. *Journal of child psychology and psychiatry, and allied disciplines.* 2000;41(5):645-655.
5. Horwood J, Salvi G, Thomas K, et al. IQ and non-clinical psychotic symptoms in 12-year-olds: results from the ALSPAC birth cohort. *The British journal of psychiatry : the journal of mental science.* 2008;193(3):185-191.
6. Zammit S, Kounali D, Cannon M, et al. Psychotic experiences and psychotic disorders at age 18 in relation to psychotic experiences at age 12 in a longitudinal population-based cohort study. *The American journal of psychiatry.* 2013;170(7):742-750.
7. Jones HJ, Stergiakouli E, Tansey KE, et al. Phenotypic Manifestation of Genetic Risk for Schizophrenia During Adolescence in the General Population. *JAMA psychiatry.* 2016;73(3):221-228.
8. McGrath JJ, Saha S, Al-Hamzawi A, et al. The Bidirectional Associations Between Psychotic Experiences and DSM-IV Mental Disorders. *The American journal of psychiatry.* 2016;173(10):997-1006.
9. Stochl J, Khandaker GM, Lewis G, et al. Mood, anxiety and psychotic phenomena measure a common psychopathological factor. *Psychological medicine.* 2015;45(7):1483-1493.
10. Milne BJ, Moffitt TE, Crump R, Poulton R, Rutter M, Sears MR, Taylor A, Caspi A. How should we construct psychiatric family history scores? A comparion of alternative approaches from the Dunedin Family Health History Study. *Psychological medicine*. 2008;38(12):1793-802.
11. Stahl E, Forstner A, McQuillin A, et al. Genomewide association study identifies 30 loci associated with bipolar disorder. *bioRxiv.* 2017.
12. Jung T, Wickrama KAS. An introduction to latent class growth analysis and growth mixture modeling. *Social and Personality Psychology Compass.* 2008;Vol.2(1):pp.
13. Seaman SR, White IR. Review of inverse probability weighting for dealing with missing data. *Statistical methods in medical research.* 2013;22(3):278-295.
14. Rai D, Culpin I, Heuvelman H, et al. Association of Autistic Traits With Depression From Childhood to Age 18 Years. *JAMA psychiatry.* 2018;75(8):835-843.
15. Jones HJ, Heron J, Hammerton G, et al. Investigating the genetic architecture of general and specific psychopathology in adolescence. *Translational psychiatry.* 2018;8(1):145.

© 2018 Rice F et al. *JAMA Psychiatry.*
